# Supplementary material for: Inclusion of young people with disabilities in the future of work: forecasting workplace, labour market and community-based strategies through an online and accessible Delphi survey protocol
Source: BMJ Open. 2022 Jul 7;12(7):e055452. doi: 10.1136/bmjopen-2021-055452 (PMC9263911; doi:10.1136/bmjopen-2021-055452)
Supplement: Author's manuscript [file bmjopen-2021-055452.draft_revisions.pdf]

BMJ Open

BMJ Open is committed to open peer review. As part of this commitment we make the peer review history of every article we publish publicly available.

When an article is published we post the peer reviewers' comments and the authors' responses online. We also post the versions of the paper that were used during peer review. These are the versions that the peer review comments apply to.

The versions of the paper that follow are the versions that were submitted during the peer review process. They are not the versions of record or the final published versions. They should not be cited or distributed as the published version of this manuscript.

BMJ Open is an open access journal and the full, final, typeset and author-corrected version of record of the manuscript is available on our site with no access controls, subscription charges or pay-per-view fees (http://bmjopen.bmj.com).

If you have any questions on BMJ Open's open peer review process please email info.bmjopen@bmj.com

BMJ Open

Inclusion of young people with disabilities in the future of work: Forecasting workplace, labor market and community-based strategies through an online and accessible Delphi survey methodology

Journal:	*BMJ Open*
Manuscript ID	bmjopen-2021-055452
Article Type:	Protocol
Date Submitted by the Author:	13-Jul-2021
Complete List of Authors:	Jetha, Arif; Institute for Work & Health; University of Toronto<br>Nasir, Kay<br>Van Eerd, Dwayne; Institute for Work & Health<br>Gignac, M; Institute for Work & Health; University of Toronto<br>Ginis, Kathleen; University of British Columbia, Department of Medicine; University of British Columbia<br>Tompa, Emile; Institute for Work & Health; McMaster University
Keywords:	OCCUPATIONAL & INDUSTRIAL MEDICINE, SOCIAL MEDICINE, Health policy < HEALTH SERVICES ADMINISTRATION & MANAGEMENT

SCHOLARONE™
Manuscripts

RUNNING HEAD: INCLUSION IN THE FUTURE OF WORK

PROTOCOL PAPER: BMJ Open

Title: Inclusion of young people with disabilities in the future of work: Forecasting workplace, labor market and community-based strategies through an online and accessible Delphi survey methodology

Authors:

Arif Jetha (ajetha@iwh.on.ca)[1,2*] Kay Nasir (knasir@iwh.on.ca), Dwayne Van Eerd (dvaneerd@iwh.on.ca) [1], Monique AM Gignac (mgignac@iwh.on.ca) [1,2], Kathleen Martin Ginis (kathleen_martin.ginis@ubc.ca)[3,4,5], Emile Tompa (etompa@iwh.on.ca)[1, 2, 6]

Affiliations:

[1] Institute for Work & Health, Toronto, ON, Canada

[2] Dalla Lana School of Public Health, University of Toronto, Toronto, ON, Canada

[3]Department of Medicine, Division of Physical Medicine & Rehabilitation, University of British Columbia, Vancouver, BC, Canada

[4]School of Health and Exercise Sciences, University of British Columbia, Kelowna, BC, Canada

[5]Centre for Chronic Disease Prevention and Management, Southern Medical Program, University of British Columbia, Kelowna, Canada

[6]McMaster University, Hamilton, ON, Canada

***Corresponding Author**

Arif Jetha

Institute for Work & Health

Suite 1800, 480 University Avenue

Toronto, ON, Canada

M5A 1S5

ajetha@iwh.on.ca

416 927 2027 ext. 2229

For peer review only

RUNNING HEAD: INCLUSION IN THE FUTURE OF WORK

Total Words = 3729

Abstract words = 280

Number of tables = 0

Acknowledgements

We would like to acknowledge Ali Shamaee for their support with study conceptualization.

We'd also like to thank our study advisory panel consisting of young adults with the lived experience of a disability. We also like to acknowledge the support of our community partners including Canadian Council on Rehabilitation and Work, Abilities Centre, and National Educational Association of Disabled Students.

For peer review only

RUNNING HEAD: INCLUSION IN THE FUTURE OF WORK

ABSTRACT

Introduction: The future of work is expected to transform the nature of work, create unique employment barriers for young people living with disabilities and disrupt pathways to better health. We present a Delphi survey through which we aim to obtain future-oriented strategies that can improve the accessibility and inclusion of young people with disabilities in the future of work.

Methods and analysis: The Delphi survey will be conducted primarily online, over two rounds and in a format that is accessible to people living with disabilities. A diverse sample of subject matter experts (e.g., policy makers, employment service providers, labor market experts) and participants with the lived experience of a disability will be recruited using a purposive sampling strategy. All participants will be asked to complete both rounds of the Delphi survey. In the first round, open-ended questions will be asked about workplace, community-based or policy supports that can support the inclusion of young people with disabilities and addresses specific future of work trends which span sociopolitical, economic, environmental and technological domains. In the second round of the survey we will aim to build consensus; participants will be provided with a summary of themes emerging from round 1 and will be asked to rate their level of agreement. Following the completion of the second round, consensus-based and future-focused recommendations will be generated that can support young people with disabilities in the world of work of the future.

For peer review only - http://bmjopen.bmj.com/site/about/guidelines.xhtml

RUNNING HEAD: INCLUSION IN THE FUTURE OF WORK

Ethics and dissemination: Study protocol approved by University of Toronto's research ethics board (#40727). The study will identify future-focused support strategies that will be shared with people living with disabilities, policy makers and disability employment service through an integrated knowledge transfer and exchange approach.

KEYWORDS

Disability, future of work, changing nature of work, inclusion, accessibility, Delphi survey, consensus, accommodation, disability policy

For peer review only

RUNNING HEAD: INCLUSION IN THE FUTURE OF WORK

STRENGTHS AND LIMITATIONS OF THIS STUDY

- Social, technological, envionrmental and economic changes that characterize the future of work are expected to reinforce and widen labor market inequities for young people with disabilities and disrupt the pathways to health
- The Delphi survey protocol will generate future-focused recommendations that can support the sustained involvement of young people with disabilities within the future of work
- The Delphi survey takes a structured and multistaged approach to collect anonymous expert opinions and feedback, facilitate unbiased debate with the goal of reaching consensus on strategies to support the employment of people with disabilities
- Generating employment support strategies for young people with disabilities from a future-facing perspective could be difficult for study participants and represents a limitation

For peer review only

For peer review only - http://bmjopen.bmj.com/site/about/guidelines.xhtml

RUNNING HEAD: INCLUSION IN THE FUTURE OF WORK

BACKGROUND

In the future, work is expected to be transformed in terms of its nature and availability, which is expected to further exacerbate employment challenges for labor market groups who have traditionally faced disadvantage [1]. Young people living with disabilities are one specific group who face difficulties entering and advancing within the world of work and benefit from strategies that promote inclusion [2 3]. Indeed, changes in work will necessitate the redesign and innovation of policies and programs that can be implemented within workplace, community or policy settings that support the engagement of young people with disabilities in employment.

Research on industrialized countries indicates that young people with disabilities face challenges finding and sustaining employment. Data from Canada, where the protocol will be implemented, indicate that the employment rate of youth (18-24 years) and young adults (25-35 years) living with a disability is significantly lower than their healthy counterparts (32% and 54% compared to 52% and 82%, respectively) [2 4]. Young people with disabilities who find paid employment are more likely to report fewer work hours, lower income, lost productivity and barriers to career advancement than their non-disabled peers [4 5]. Of concern, challenges faced by

For peer review only

young people with disabilities during the early career phase can have a lasting effect and contribute to adverse labor market outcomes that extend across the working life course [6].

Improving accessibility and inclusion of young people with disabilities in paid employment represents an important organizational, social policy and public health priority. Studies indicate strategies that address accessibility to the physical work environment, strengthen interpersonal relationships, address discrimination, encourage job autonomy and facilitate healthcare access are important to the employment of people with disabilities [5 7-9]. The absence of these strategies can contribute to missed workdays, reduced work hours and greater workplace activity limitations. To date, research on the accessible and inclusive employment of young people with disabilities entering and advancing in the world of work have been developed within existing labor market contexts but they may not account for potential changes to work that are expected to occur in the future.

It is anticipated that in the future, work will be characterized by interrelated social, technological, environmental, economic and political driving forces that are expected to disrupt every industry, transform working conditions and affect the types and availability of jobs [1]. The rapid digital transformation of the economy is considered a defining feature of the future of work. The development and application of diverse digital technologies – such as artificial

For peer review only - http://bmjopen.bmj.com/site/about/guidelines.xhtml

intelligence (AI) and machine learning systems, advanced robots, smart sensors, Internet of Things, blockchain technology or self-driving vehicles – are occurring at a faster pace compared to past periods of technological adoption and are resulting in significant change to the world of work [10-13]. A recent synthesis of literature on the future of work noted that it will also be characterized by social (e.g., generational changes in the workforce), political (e.g., growing populist sentiments) and environmental changes (e.g., climate change) that can create opportunities and challenges for workers [1].

It is anticipated that changes in work will have the potential to contribute to productivity, create new occupations and enable new working arrangements in ways that can increase employment opportunities and demand for diverse workers [14]. At the same time, studies highlight that the future of work could be marked by widening labor market inequity such as an more rapid erosion of standard employment opportunities (e.g., full-time permanent jobs) and growth of non-traditional employment (e.g., freelancing, gig work) coupled with wage stagnation [14]. Workers in lower-skilled jobs could be in a position of disadvantage and be susceptible to disruption in the future [1 12 15]. The growing application of machine learning within workplaces

For peer review only

enable the automation of increasingly complex and predictive job tasks and contribute to the displacement of workers in higher-skilled and professional occupations [12].

Inclusive strategies are required for young people with disabilities within the future of work that provide protection from potential disruptions and so that young people with disabilities can access potential opportunities. The integration of young people with disabilities in the future of work has the potential to strengthen longer-term socioeconomic pathways to better health and quality of life [16 17]. What is more, strategiges that promote the employment of people with disabilities have broader societal (e.g., decreased demand for income support) and commercial benefits (e.g., greater access to a highly skilled and motiviated labor market group) [18].

We describe an online Delphi survey protocol that aims to collect insight from diverse experts on the strategies that can support the sustained involvement of young people with disabilities within the future of work. Our study protocol has three specific objectives:

1. Identify and build consensus on the specific workplace supports, policies or community-based programs for young people with disabilities that can foster inclusion in the futureof work.
2. Determine the barriers that young people with disabilities could encounter to accessing workplace supports, policies or community-based programs within the context of work in the future.

For peer review only - http://bmjopen.bmj.com/site/about/guidelines.xhtml

3. Design concrete recommendations for employers, policymakers and community-based organizations to promote inclusion of young people with disabilities in the future of work.

METHODS AND ANALYSIS

Context

Our protocol will be implemented in Canada, where the federal government recently passed the Accessible Canada Act (ACA), legislation that has employment as one of seven priority areas. The ACA legislates the creation of a barrier-free Canada through proactive identification, prevention and removal of barriers to accessibility in federally regulated workplaces [19]. Passing the ACA has moved Canada closer to meeting its human rights and equity obligations under the United Nations Convention on the Rights of Persons with Disabilities to which it is a signatory[20]. To successfully ensure accessibility within work environments for people with disabilities and achieve the legislative goals of the ACA, strategic planning is required to anticipate potential barriers that could emerge within the context of the changing nature of work.

Our Delphi survey also emerges from a large-scale evidence synthesis of the future of work that may contribute to employment inequities [1]. The synthesis uncovered nine key future of

For peer review only - http://bmjopen.bmj.com/site/about/guidelines.xhtml

work trends: the digital transformation of the economy, artificial intelligence (AI) and machine learning-enhanced automation, AI-enabled human resource management systems, skill requirements for the future of work, globalization 4.0, climate change and the green economy, Gen Zs and the work environment, populism and the future of work, and external shocks to accelerate the changing nature of work. The study also found that these trends were interrelated and could individually and collectively contribute to barriers and opportunities for young people with disabilities in the working world. The Delphi survey we describe will enable participants to generate strategies that specifically address the nine future of work trends that were identified in the recent evidence synthesis [1].

Conceptual approach and Delphi survey overview

Our protocol draws on methods from the field of strategic foresight to obtain future-oriented strategies that can support the employment of young people with disabilities within the context of the future of work. One approach commonly used in strategic foresight is a Delphi survey to obtain insights on uncertain and complex phenomena and develop related solutions. The Delphi survey is a participatory process which draws on the knowledge of diverse experts [21 22]. The Delphi survey takes a structured and multistage approach to collect anonymous expert opinions

For peer review only - http://bmjopen.bmj.com/site/about/guidelines.xhtml

and feedback, facilitate unbiased debate and reach consensus on a phenomenon with the ultimate goal of generating future-oriented recommendations that contribute to policy and programmatic development [22]. The Delphi survey was initially developed by the RAND Corporation in the 1950s for technological forecasting in US military research applications [22] but has been increasingly been applied to policy and programmatic development in a range of sectors including technology, finance, education, and health [23-26]. More recently, online Delphi surveys have become more common and enable participants to complete the study at their own time and pace and enable researchers to collect insights from diverse geographical locations. An online Delphi survey format also ensures anonymity of participants to address biases that emerge in traditional forms of in-person group decision-making and can ensure that data collection is accessible to participants with disabilities and the assistive devices they may require (e.g., completion of Delphi survey using screen readers) [23 27-30].

Sample recruitment

A key element in the design and implementation of a Delphi survey is the selection of Canadian and international participants with subject matter expertise on the employment of young people with disabilities or the changing world of work and/or the lived experience of a disability [31]. Subject matter experts recruited for this study will include policymakers, disability employment service providers, educators, clinicians and futurists. All eligible participants should be above 18

For peer review only - http://bmjopen.bmj.com/site/about/guidelines.xhtml

years of age, willing to contribute to structured online discussions on the design of innovative strategies to support the employment of young people with disabilities within the context of different future of work trends.

Recruitment will be purposive to ensure we capture a wide range of expertise. Participants will be recruited with the support of our advisory panel and research colleagues who will send direct study invitations to eligible subject matter experts and people with the lived experience of a disability. Additionally, recruitment information will be shared via social media and listservs maintained by the national disability organizations to which our advisory panel are representatives. We will take a snowball approach to recruitment. Participants who complete the Delphi survey will be asked to nominate other potential participants who may be contacted. Currently no established guidelines exist on the number of participants required for a Delphi survey. Researchers utilizing a Delphi approach suggest that sample sizes should be large enough to capture breadth and depth in perspectives but also be manageable for the research team to enable retention across multiple survey rounds. Aligning with past Delphi studies of a similar nature to our protocol [32], we aim to recruit up to 200 participants to enable descriptive analyses required to establish consensus and address potential challenges related to following up with participants [33].

Survey design

The Delphi survey will be implemented in at least two rounds in English and French languages. According to past applications of Delphi surveys, two rounds can be optimal because it limits

For peer review only - http://bmjopen.bmj.com/site/about/guidelines.xhtml

participant fatigue and dropout which may bias results [34]. The survey was developed based on multiple stages of pilot testing with members of our advisory panel. Pilot testing ensured face validity of questions, use of comprehensible language and that the survey could be completed within 20 minutes. Also, pilot testing ensured that the format was inclusive for participants with different disabilities and could be used in conjunction with assistive devices (e.g., screen readers).

Delphi survey items are developed to foster virtual brainstorming and debate and capture participant insights on specific strategies that can support the employment of young people with disabilities and are relevant to different trends in the future of work. Prompts are included throughout the survey to encourage participants to think strategically about the future and consider how changing conditions could impact young people with disabilities and their need for workplace, community-based and policy supports. The first survey round focuses specifically on idea generation and obtaining specific strategies that can support entry and advancement of young people with disabilities in the future of work [35]. Participants will be presented with a description of the trend and provided with additional reading where they can learn more on the topic if they require. A series of questions corresponding to the specific trend will be asked. Participants will be asked to think about the importance of the trend to shaping the long-term employment of people with disabilities. Also, participants will be asked about whether strategies can be implemented within the community, workplace or at the policy-level to address the impact of the trend on the employment of young people with disabilities. An open-ended question will ask participants to describe specific strategies that correspond to their previous answers. A final open-ended question will be presented to a participant where they can explain

For peer review only

For peer review only - http://bmjopen.bmj.com/site/about/guidelines.xhtml

the rationale behind their responses. Participants will be randomly allocated four out of the nine future of work trends identified from past research.

All open-ended questions from the first round of the Delphi survey will be coded using thematic analysis and an iterative approach. Survey responses will be reviewed by members of the research team, and a codebook will be developed through several conversations. The codebook will be applied to analyze Delphi survey responses. A primary and secondary coder will conduct a line-by-line coding of responses. Throughout the analytical process, codes and themes emerging from the Delphi survey will be discussed in research team analysis meetings and any inconsistencies will be resolved by consensus. Through our analytical process, a standardized list of strategies for young people with disabilities that are relevant to each trend in the future of work will be generated accordingly. A summary of findings will be integrated into a short report. The study team will ensure that findings are presented anonymously so that participant responses are not biased in subsequent phases of the Delphi survey [36].

The second round of the Delphi survey, administered after six months, aims to reach consensus across study participants. All participants will be asked to read the brief report that will include findings from the previous round that are aggregated across all study participants as well as a summary of the participant's specific responses. Participants will be asked to complete the second round of the survey while considering findings from the first round. Participants will be presented with the same four trends that they were presented with in round one and the corresponding strategies that were generated. Participants will be asked to rate their level of agreement with the specific strategies that have been proposed for each trend using Likert-style

For peer review only - http://bmjopen.bmj.com/site/about/guidelines.xhtml

questions (e.g., 1=not at all agree; 5=extremely agree). Additionally, open-ended questions will ask about anticipated barriers in implementing strategies to addressing the future of work trend.

The results of the second Delphi survey will be summarized using descriptive statistics [22]. There exist no widely used recommendations on cut-off points that can be used by research to establish consensus in a Delphi survey. Aligning with past applications of the Delphi survey approach, consensus will be reached if at least 70% of participants report agreement on the ability of specific strategy to address a future of work trend [32]. Open-ended questions will be thematically coded using the same iterative approach that is taken in the analysis of findings in Delphi survey round 1. Following the completion of the second round of the Delphi survey, we anticipate having generated consensus-based policy and programmatic recommendations that can support young people with disabilities that are relevant to the future of work. We will also be able to obtain specific insights into the obstacles that may be faced in implementing and accessing the proposed strategies.

Patient and Public Involvement

Our study takes an integrated knowledge transfer and exchange approach (iKTE) which is defined as a process of two-way exchange between researchers and diverse stakeholders at all phases of the research process including study design and implementation, analysis and synthesis and dissemination to increase research relevancy and evidence uptake and application [37-39]. To implement an iKTE approach in our protocol, we established an advisory panel consisting of young people with lived experience of disability [39] and representatives from national non-profit disability organizations. Advisory panel members have been engaged to inform study design and recruitment and accessibility of data collection approaches. Following the administration of the

For peer review only - http://bmjopen.bmj.com/site/about/guidelines.xhtml

Delphi survey, the advisory panel will be asked to help interpret findings and co-create knowledge mobilization outputs.

Ethics and dissemination

The research has been cleared by the University of Toronto's Research Ethics Board (#40727). All data collection and analysis will be performed in accordance with research ethics guidelines and regulations for research on human subjects. Of note, all participants will be presented with detailed study material and informed consent will be obtained prior to their participation in the study. Honorariums will be provided to participants with lived experience. All data we collect will be anonymized and stored on a secure server. Our iKTE approach will produce findings that are more relevant to the unique lived experience of young people with disabilities and useable to stakeholders to inform strategies for the future of work [38]. In collaboration with our advisory panel we will design outputs that are tailored and shared to dirverse knowledge users including policy makers, disability employment service providers, employers and people living with disabilities.

DISCUSSION

Young people with disabilities represent a vulnerable labor market subgroup who may require strategies to address the challenges associated with entering and advancing within the world of work. In the future, work is expected to shift the nature and availability of work and, as a result,

For peer review only - http://bmjopen.bmj.com/site/about/guidelines.xhtml

create new barriers and facilitators to employment participation. The Delphi survey methodology that we present has the potential to contribute to future-oriented insights regarding the specific strategies that can be used by employers, policymakers and employment service providers to promote the employment of people with disabilities that are resilient to the changing nature of work. Importantly, the strategies produced through the Delphi survey have the potential to strengthen present and future socioeconomic pathways to health and quality of life of young people with disabilities [40].

Past studies of young people with disabilities highlight the importance of diverse support strategies to foster employment and career advancement including workplace practices (e.g., job accommodation), labor market policies (e.g., anti-discrimination laws) and community-based programs (e.g., work-integrated learning) [3 9]. Yet, social, political, technological and environmental changes in the future of work will drive changes to workplace and labor market conditions [1 13]. As a result, existing support strategies for people with disabilities will require innovation. In this paper, we describe a Delphi survey protocol which is a well-suited methodology to undertake exploratory research to develop insights on the disability support strategies in the future of work. Past scholars note that Delphi surveys have the advantage of

For peer review only - http://bmjopen.bmj.com/site/about/guidelines.xhtml

being a rapid approach to collecting and combining expertise from diverse subject matter experts across geographic regions [32]. A Delphi approach may not replace methods which establish causation. Intervention research will be required to test the support strategies uncovered in the Delphi study in terms of their ability to foster the long-term employment of young people with disabilities.

A key objective of this Delphi survey will be to facilitate the convergence of future-focused perspectives among participants and forecast support strategies [41]. By administering the survey over two or more rounds, participants will be exposed to the responses of others. In doing so, they will explore their underlying assumptions regarding the future of work which may have informed their responses and compare their perspectives to those held by other subject matter experts [22]. While the focus of our Delphi approach is to establish consensus on future of work support strategies, we will also review strategies that have been proposed where there is a lack of consensus [34]. Additional follow-up research may be required to examine the potential implications of strategies that are recommended by participants and where consensus is not reached to determine utility towards supporting young people with disabilities

Research and practice on the employment of people with disabilities highlight the importance of that multiple perspectives are necessary to identify different barriers and

For peer review only

facilitators to participation in paid work and develop corresponding solutions [42]. We will recruit policymakers from different levels of government and labor market specialists and employment service providers from across Canada to capture a range of insights regarding the strategies that correspond to different changes in the future of work. Drawing on a diversity of insights can contribute to the development of future-focused wraparound supports which are seen as an important strategy to foster the employment of people with disabilities. Moreover, disability scholars highlight an imperative of ensuring the lived experience to predominate in research on people with disabilities [42 43]. Our study takes an iKTE approach; we created an advisory panel where we integrate lived experience into study design and implementation, data analysis and knowledge mobilization. Through our iKTE approach we will produce findings that are applicable to diverse knowledge users and can be implemented to address different challenges faced by young people with disabilities in the future of work [38 39]. We anticipate that our findings will also have indirect outcomes for advisory panel members and study participants. By participating in the Delphi survey, participants will be encouraged to take a future-focused perspective towards understanding the employment challenges and opportunities faced by young people with disabilities and take a more proactive approach to designing support strategies [44].

For peer review only - http://bmjopen.bmj.com/site/about/guidelines.xhtml

There are some limitations of our Delphi survey. First, for subject matter experts, thinking about work-related challenges and opportunities for people with disabilities and generating support strategies from a future-facing perspective could be difficult. To address this challenge, we have included prompts throughout the survey that encourage participants to consider the relevancy of their responses for the future. Secondly, consistent with past applications of a Delphi survey approach, we will take a purposive sampling strategy to identify diverse subject matter experts and people with the lived experience of a disability. While representativeness of the sample is not a primary goal, we will take steps to ensure depth and breadth in survey responses and the development of recommendations to support young people with disabilities in the future of work [36]. Third, loss to follow-up represents a barrier to establishing consensus and could be a source of bias. Although time and resource intensive, members of the research team will take an active approach to engaging and following up with all participants in the second phase of the Delphi survey to address the potential for dropout.

Conclusion

For young people with disabilities, employment represents a critical social determinant of health and quality of life. Yet, many young people report challenges as they enter and advance within

For peer review only - http://bmjopen.bmj.com/site/about/guidelines.xhtml

the working world. We describe a Delphi survey approach to generate recommendations and build consensus on future-focused employment support strategies drawn form a diverse group of subject matter experts and people with lived experience of a disability. The evidence to be generated from our study can be used to inform the design of workplace, community-based and policy-level strategies to ensure that the work in the future will include young people with disabilities.

AUTHOR CONTRIBUTIONS

Arif Jetha: Conceptualization, Methodology, Writing - Original Draft, Funding acquisition

Kay Nasir: Conceptualization, Methodology, Writing - Original Draft

Dwayne Van Eerd: Conceptualization, Methodology, Writing – Review and Editing

Monique Gignac: Conceptualization, Methodology, Writing – Review and Editing

Kathleen Martin Ginis: Conceptualization, Methodology

Emile Tompa: Conceptualization, Methodology, Writing – Review and Editing

FUNDING

For peer review only - http://bmjopen.bmj.com/site/about/guidelines.xhtml

RUNNING HEAD: INCLUSION IN THE FUTURE OF WORK

This work was supported by a research operating grant from Accessibility Standards Canada, Employment and Social Development Canada, Government of Canada (#017323700). The study protocol has undergone peer-review by the funding body. Funding bodies had no role in study design and will not be involved in data collection, data interpretation or manuscript writing.

COMPETING INTERESTS

No authors have competing interests to report related to this research.

AVAILABILITY OF DATA AND MATERIALS

The datasets generated and analyzed in the current study will not be publicly available to protect the privacy of research participants as outlined in the study's research ethics protocol. Those with data requests may contact the corresponding author to learn more about why data cannot be released.

REFERENCES

1. Jetha A, Shamaee A, Bonaccio S, et al. Fragmentation in the future of work: A horizon scan examining the impact of the changing nature of work on workers experiencing vulnerability. *American Journal of Industrial Medicine* 2021;In press

For peer review only

2. Morris SP, Fawcett G, Brisebois L, et al. A demographic, employment and income profile of Canadians with disabilities aged 15 years and over, 2017. Ottawa, ON: Statistics Canada, 2018.

3. Jetha A, Bowring J, Furrie A, et al. Supporting the transition into employment: a study of Canadian young adults living with disabilities. *Journal of occupational rehabilitation* 2019;29(1):140-49.

4. Statistics Canada. A profile of persons with disabilities among Canadians aged 15 years or older, 2012. Ottawa, ON, 2017. Contract No. 89-654-X.

5. Jetha A, Tucker L, Backman C, et al. Rheumatic disease disclosure at the early career phase and its impact on the relationship between workplace supports and presenteeism. *Arthritis Care & Research* 2021 doi: https://doi.org/10.1002/acr.24620

6. Scarpetta S, Sonnet A, Manfredi T. Rising youth unemployment during the crisis: How to prevent negative long-term consequences on a generation? . *OECD Social, Employment and Migration Working Papers,* 2010; (No.6. 2010).

For peer review only - http://bmjopen.bmj.com/site/about/guidelines.xhtml

7. Nevala N, Pehkonen I, Koskela I, et al. Workplace accommodation among persons with disabilities: A systematic review of its effectiveness and barriers or facilitators. *Journal of occupational rehabilitation* 2015;25(2):432-48.

8. Padkapayeva K, Posen A, Yazdani A, et al. Workplace accommodations for persons with physical disabilities: evidence synthesis of the peer-reviewed literature. *Disability and rehabilitation* 2017;39(21):2134-47.

9. Jetha A, Shaw R, Sinden AR, et al. Work-focused interventions that promote the labour market transition of young adults with chronic disabling health conditions: a systematic review. *Occupational and environmental medicine* 2019;76(3):189-98.

10. Baldwin R. The globotics upheaval: Globalization, robotics, and the future of work. New York: Oxford University Press 2019.

11. Manyika J, Chui M, Miremadi M, et al. A future that works: AI, automation, employment, and productivity. (p. 28), 2017.

12. Acemoglu D, Restrepo P. Robots and jobs: Evidence from US labor markets. *Journal of Political Economy* 2020;128(6):2188-244.

For peer review only

For peer review only - http://bmjopen.bmj.com/site/about/guidelines.xhtml

13. World Economic Forum. The future of jobs report 2020. Geneva: World Economic Forum., 2021.

14. Autor D, Mindell D, Reynolds E. The work of the future: Building better jobs in an age of intelligent machines, 2020.

15. Acemoglu D, Restrepo P. Low-skill and high-skill automation. *Journal of Human Capital* 2018;12(2):204-32.

16. Benach J, Benavides FG, Platt S, et al. The health-damaging potential of new types of flexible employment: a challenge for public health researchers. *American journal of public health* 2000;90(8):1316-7. doi: 10.2105/ajph.90.8.1316 [published Online First: 2000/08/11]

17. Mann DR, Wittenburg DC. Starting behind: Wage and employment differentials between young adults with and without disabilities. *Journal of Disability Policy Studies* 2015;26(2):89-99.

18. Bonaccio S, Connelly CE, Gellatly IR, et al. The participation of people with disabilities in the workplace across the employment cycle: Employer concerns and research evidence. *Journal of Business and Psychology* 2020;35(2):135-58.

For peer review only

19. An act to ensure a barrier-free Canada. Bill C-81, 2018.

20. United Nations. Convention on the Rights of Persons with Disabilities (CRPD), 2019.

21. Mozuni M, Jonas W. An introduction to the morphological Delphi method for design: A tool for future-oriented design research. *She Ji: The Journal of Design, Economics, and Innovation* 2017;3(4):303-18.

22. Hasson F, Keeney S, McKenna H. Research guidelines for the Delphi survey technique. *Journal of advanced nursing* 2000;32(4):1008-15.

23. Aichholzer G. The delphi method: Eliciting experts' knowledge in technology foresight. Interviewing experts: Springer 2009:252-74.

24. Kozak M, Iefremova O. Implementation of the Delphi technique in finance. *e-Finanse: Financial Internet Quarterly* 2014;10(4):36-45.

25. Nworie J. Using the Delphi technique in educational technology research. *TechTrends* 2011;55(5):24-30.

26. Beech B. The Delphi approach: recent applications in health care. *Nurse Researcher (through 2013)* 2001;8(4):38.

For peer review only

For peer review only - http://bmjopen.bmj.com/site/about/guidelines.xhtml

27. Hong SG, Trimi S, Kim DW, et al. A Delphi study of factors hindering web accessibility for persons with disabilities. *Journal of Computer Information Systems* 2015;55(4):28-34.

28. Helmer-Hirschberg O. Analysis of the future: The Delphi method. Santa Monica: Rand Corporation 1967.

29. Revez A, Dunphy N, Harris C, et al. Beyond Forecasting: Using a Modified Delphi Method to Build Upon Participatory Action Research in Developing Principles for a Just and Inclusive Energy Transition. *International Journal of Qualitative Methods* 2020;19 doi: https://doi.org/10.1177/1609406920903218

30. Dayé C. How to train your oracle: The Delphi method and its turbulent youth in operations research and the policy sciences. *Social Studies of Science* 2018;48(6):846-68.

31. Belton I, MacDonald A, Wright G, et al. Improving the practical application of the Delphi method in group-based judgment: a six-step prescription for a well-founded and defensible process. *Technological Forecasting and Social Change* 2019;147:72-82.

32. Hsu C-C, Sandford BA. The Delphi technique: making sense of consensus. *Practical Assessment, Research, and Evaluation* 2007;12(1):10.

For peer review only - http://bmjopen.bmj.com/site/about/guidelines.xhtml

33. Keller J, Heiko A. The influence of information and communication technology (ICT) on future foresight processes—Results from a Delphi survey. *Technological Forecasting and Social Change* 2014;85:81-92.

34. Gossler T, Sigala IF, Wakolbinger T, et al. Applying the Delphi method to determine best practices for outsourcing logistics in disaster relief. *Journal of Humanitarian Logistics and Supply Chain Management* 2019;9(3):438-74.

35. Arif Jetha AS. Fragmentation in the future of work. Nine trends that may shape the future of work and what they mean for vulnerable workers, 2021.

36. Asselin M, Harper M. Revisiting the Delphi technique: implications for nursing professional development. *Journal for Nurses in Professional Development* 2014;30(1):11-15.

37. Dew A, Boydell KM. Knowledge translation: Bridging the disability research-to-practice gap. *Research and Practice in Intellectual and Developmental Disabilities* 2017;4(2):142-57.

38. Rogers JD, Martin FH, Force NKTT. Knowledge translation in disability and rehabilitation research: lessons from the application of knowledge value mapping to the case of accessible currency. *Journal of Disability Policy Studies* 2009;20(2):110-26.

For peer review only

39. Van Eerd D, Saunders R. Integrated knowledge transfer and exchange: An organizational approach for stakeholder engagement and communications. *Scholarly and Research Communication* 2017;8(1)

40. Ahonen EQ, Fujishiro K, Cunningham T, et al. Work as an inclusive part of population health inequities research and prevention. *American journal of public health* 2018;108(3):306-11.

41. US Department of Health and Human Services Public Health Services Agency for Health Care Policy and Research. Acute Pain Management: Operative or Medical Procedures and Trauma. . Rockville: Acute Pain Management Guideline Panel 1992.

42. Bonaccio S, Connelly CE, Fisher SL, et al. Beyond managing research partnerships: Partnered research as an integrated methodological approach. *Industrial and Organizational Psychology* 2018;11(4):613-19.

43. Harpur P. Nothing about us without us: The UN convention on the rights of persons with disabilities. Oxford Research Encyclopedia of Politics2017.

44. van der Heijden K. Scenarios, strategy and the strategy process. *Global Business Network Presearch, Provoking Strategic Conversation* 1997;1(1)

For peer review only

For peer review only - http://bmjopen.bmj.com/site/about/guidelines.xhtml

For peer review only

BMJ Open

Inclusion of young people with disabilities in the future of work: Forecasting workplace, labor market and community-based strategies through an online and accessible Delphi survey protocol

Journal:	*BMJ Open*
Manuscript ID	bmjopen-2021-055452.R1
Article Type:	Protocol
Date Submitted by the Author:	08-Dec-2021
Complete List of Authors:	Jetha, Arif; Institute for Work and Health; University of Toronto<br>Nasir, Kay; Institute for Work and Health<br>Van Eerd, Dwayne; Institute for Work & Health<br>Gignac, M; Institute for Work & Health; University of Toronto<br>Ginis, Kathleen; University of British Columbia, Department of Medicine; University of British Columbia<br>Tompa, Emile; Institute for Work & Health; McMaster University
**Primary Subject Heading**:	Occupational and environmental medicine
Secondary Subject Heading:	Public health, Research methods, Rehabilitation medicine, Qualitative research
Keywords:	OCCUPATIONAL & INDUSTRIAL MEDICINE, SOCIAL MEDICINE, Health policy < HEALTH SERVICES ADMINISTRATION & MANAGEMENT

SCHOLARONE™
Manuscripts

RUNNING HEAD: INCLUSION IN THE FUTURE OF WORK

PROTOCOL PAPER: BMJ Open

Title: Inclusion of young people with disabilities in the future of work: Forecasting workplace, labor market and community-based strategies through an online and accessible Delphi survey protocol

Authors:

Arif Jetha (ajetha@iwh.on.ca)[1,2*] Kay Nasir (knasir@iwh.on.ca)[1,2], Dwayne Van Eerd (dvaneerd@iwh.on.ca) [1], Monique AM Gignac (mgignac@iwh.on.ca) [1,2], Kathleen Martin Ginis (kathleen_martin.ginis@ubc.ca)[3,4,5], Emile Tompa (etompa@iwh.on.ca)[1, 2, 6]

Affiliations:

[1] Institute for Work & Health, Toronto, ON, Canada

[2] Dalla Lana School of Public Health, University of Toronto, Toronto, ON, Canada

[3]Department of Medicine, Division of Physical Medicine & Rehabilitation, University of British Columbia, Vancouver, BC, Canada

[4]School of Health and Exercise Sciences, University of British Columbia, Kelowna, BC, Canada

[5]Centre for Chronic Disease Prevention and Management, Southern Medical Program, University of British Columbia, Kelowna, Canada

[6]McMaster University, Hamilton, ON, Canada

***Corresponding Author**

Arif Jetha

Institute for Work & Health

Suite 1800, 480 University Avenue

Toronto, ON, Canada

M5A 1S5

ajetha@iwh.on.ca

416 927 2027 ext. 2229

For peer review only

RUNNING HEAD: INCLUSION IN THE FUTURE OF WORK

Total Words = 4,221

Abstract words = 281

Number of tables = 0

Acknowledgements

We would like to acknowledge Ali Shamaee for their support with study conceptualization.

We'd also like to thank our study advisory panel consisting of young adults with the lived experience of a disability. We also like to acknowledge the support of our community partners including Canadian Council on Rehabilitation and Work, Abilities Centre, and National Educational Association of Disabled Students.

For peer review only

RUNNING HEAD: INCLUSION IN THE FUTURE OF WORK

ABSTRACT

Introduction: The future of work is expected to transform the nature of work, create unique employment barriers for young people living with disabilities and disrupt pathways to better health. We present a Delphi survey protocol through which we aim to obtain future-oriented strategies that can improve the accessibility and inclusion of young people with disabilities in the future of work.

Methods and analysis: The Delphi survey will be conducted primarily online, over two rounds and in a format that is accessible to people living with disabilities. A diverse sample of subject matter experts (e.g., policy makers, employment service providers, labor market experts) and participants with the lived experience of a disability will be recruited using a purposive sampling strategy. All participants will be asked to complete both rounds of the Delphi survey. In the first round, open-ended questions will be asked about workplace, community-based or policy supports that can support the inclusion of young people with disabilities and addresses specific future of work trends which span sociopolitical, economic, environmental, and technological domains. In the second round of the survey, we will aim to build consensus; participants will be provided with a summary of themes emerging from round 1 and will be asked to rate their level of agreement. Following the completion of the second round, consensus-based and future-focused recommendations will be generated that can support young people with disabilities in the world of work of the future.

For peer review only

RUNNING HEAD: INCLUSION IN THE FUTURE OF WORK

Ethics and dissemination: Study protocol approved by University of Toronto's research ethics board (#40727). The study will identify future-focused support strategies that will be shared with people living with disabilities, policy makers and disability employment service through an integrated knowledge transfer and exchange approach.

KEYWORDS

Disability, future of work, changing nature of work, inclusion, accessibility, Delphi survey, consensus, accommodation, disability policy

For peer review only

RUNNING HEAD: INCLUSION IN THE FUTURE OF WORK

STRENGTHS AND LIMITATIONS OF THIS STUDY

- Through a structured approach, the Delphi survey elicits future-focused recommendations that can support the involvement of young people with disabilities within the future of work.
- The Delphi survey collects anonymous expert opinions and feedback, facilitates unbiased debate with the goal of reaching consensus on strategies to support the employment of people with disabilities.
- Multiple Delphi survey rounds, that build on one another, can help to establish consensus across participants on strategies to support the employment of young people with disabilities.
- Generating employment support strategies for young people with disabilities from a future-facing perspective could be difficult for study participants and represents a limitation.

For peer review only

RUNNING HEAD: INCLUSION IN THE FUTURE OF WORK

BACKGROUND

In the future, work is expected to be transformed in terms of its nature and availability. These changes are expected to exacerbate employment challenges for labor market groups who have traditionally faced disadvantage [1]. Young people living with disabilities are one specific group who face difficulties entering and advancing within the world of work and benefit from strategies that promote inclusion [2 3]. Indeed, changes to work necessitate the redesign and innovation of policies and programs that can be implemented within workplace, community or policy settings that support the engagement of young people with disabilities in employment.

Research on industrialized countries indicate that young people with disabilities face challenges finding and sustaining employment. Data from Canada, where the study will be conducted, indicate that the employment rate of youth (18-24 years) and young adults (25-35 years) living with a disability is significantly lower than their healthy counterparts (32% and 54% compared to 52% and 82%, respectively) [2 4]. Young people with disabilities who find paid employment are more likely to report fewer work hours, lower income, lost productivity and barriers to career advancement than their non-disabled peers [4 5]. The barriers to transitioning to employment reported by young Canadians with disabilities parallel those documented studies

For peer review only - http://bmjopen.bmj.com/site/about/guidelines.xhtml

conducted in other countries such as Australia, Sweden, and United Kingdom [6-8]. Of concern, challenges faced by young people with disabilities during the early career phase can have a lasting effect and contribute to adverse labor market outcomes that extend across the working life course [9].

Improving accessibility and inclusion of young people with disabilities in paid employment represents a priority for employers and social policy policymakers and has significant implications for public health. Strategies that address accessibility to the physical work environment, strengthen interpersonal relationships, address discrimination, encourage job autonomy and facilitate healthcare access are important to supporting the employment of people with disabilities [5 10-12]. The absence of these strategies can contribute to missed workdays, reduced work hours and greater workplace activity limitations. To date, research on the accessible and inclusive employment of young people with disabilities entering and advancing in the world of work have been developed within existing labor market contexts and may not account for potential changes to work that are expected to occur in the future.

It is anticipated that in the future, work will be characterized by interrelated social, technological, environmental, economic, and political driving forces that are expected to disrupt every industry, transform working conditions, and affect the types and availability of jobs [1 13].

For peer review only - http://bmjopen.bmj.com/site/about/guidelines.xhtml

The rapid digital transformation of the economy is considered a defining feature of the future of work. The development and application of diverse digital technologies – such as artificial intelligence (AI) and machine learning systems, advanced robots, smart sensors, Internet of Things, blockchain technology or self-driving vehicles – are occurring at a faster pace compared to past periods of technological adoption and are resulting in significant change to the world of work [14-17]. The COVID-19 pandemic has accelerated employer investment into AI applications and other digital technologies to maintain productivity amidst lockdown and to address safety concerns related to disease transmission[13 18]. A recent synthesis of literature on the future of work noted that it will also be characterized by social (e.g., generational changes in the workforce), political (e.g., growing populist sentiments) and environmental changes (e.g., climate change) that can create opportunities and challenges for workers [1].

Of note, changes to work have the potential to contribute to productivity gains, create new occupations and enable new working arrangements in ways that can increase employment opportunities and demand for diverse workers [19]. At the same time, studies highlight that the future of work could be marked by widening labor market inequity such as an more rapid erosion of standard employment opportunities (e.g., full-time permanent jobs) and growth of non-

For peer review only - http://bmjopen.bmj.com/site/about/guidelines.xhtml

traditional employment (e.g., freelancing, gig work) coupled with wage stagnation [19]. Of concern, disadvantaged groups of workers, including people living with disabilities, may be particularly susceptible to disadvantage in the future of work[1 16 20]. Other studies indicate that the growing application of advanced technologies like machine learning within workplaces can enable the automation of increasingly complex and predictive job tasks [16]. Accordingly, advanced technologies have the potential to contribute to the displacement of a broader subset of workers including those with greater educational attainment and who are employed in higher-skilled and professional occupations.

Inclusive strategies are required for young people with disabilities within the future of work that provide protection from potential disruptions and can increase access potential opportunities. Drawing on a social determinants of health framework, the integration of young people with disabilities in the future of work has the potential to strengthen longer-term socioeconomic pathways to better health and quality of life [21 22]. What is more, promoting the employment of people with disabilities have broader societal (e.g., decreased demand for income support or social assistance) as well commercial benefits (e.g., greater access to a highly skilled and motivated workforce to address labor shortages) [23]. There is a need to design strategies that can

For peer review only - http://bmjopen.bmj.com/site/about/guidelines.xhtml

ensure that the most vulnerable workers are protected from adverse changes in the future of work and are able to access potential opportunities. It is important to describe and implement a study protocol that can collect insights of diverse experts to forecast strategies that can be implemented in the present and ensure sustained employment of young people living with disabilities in the future.

We describe an application of an online accessible Delphi survey protocol that aims to collect insight from diverse experts on the strategies that can support the sustained involvement of young people with disabilities within the future of work. Our planned study has three specific objectives:

1. Identify and build consensus on the specific workplace supports, policies or community-based programs for young people with disabilities that can foster inclusion in the future of work.
2. Determine the barriers that young people with disabilities could encounter to accessing workplace supports, policies or community-based programs within the context of work in the future.
3. Design concrete recommendations for employers, policymakers, and community-based organizations to promote inclusion of young people with disabilities in the future of work.

METHODS AND ANALYSIS

For peer review only - http://bmjopen.bmj.com/site/about/guidelines.xhtml

Context

Sociopolitical and economic contextual factors play a significant role in the determining the challenges and opportunities in the future of work as well as the types of labor market policies or employment programs that can be implemented to support young people living with disabilities [13]. Our study will be implemented in Canada, where the federal government recently passed the Accessible Canada Act (ACA), legislation that has employment as one of seven priority areas. The ACA legislates the creation of a barrier-free Canada through proactive identification, prevention and removal of barriers to accessibility in federally regulated workplaces [24]. Passing the ACA has moved Canada closer to meeting its human rights and equity obligations under the United Nations Convention on the Rights of Persons with Disabilities (CRPD) to which it is a signatory[25]. To successfully ensure accessibility within work environments for people with disabilities and achieve the legislative goals of the ACA, strategic planning is required to anticipate potential barriers that could emerge within the context of the changing nature of work. Of note, findings from our study have implications for other industrialized countries, especially those who are also signatories to the CRPD[25].

For peer review only - http://bmjopen.bmj.com/site/about/guidelines.xhtml

Our Delphi survey emerges from a large-scale evidence synthesis of the future of work that may contribute to employment inequities [1]. The synthesis uncovered nine key future of work trends: the digital transformation of the economy, artificial intelligence (AI) and machine learning-enhanced automation, AI-enabled human resource management systems, skill requirements for the future of work, globalization 4.0, climate change and the green economy, GenZs and inclusive work environments, populism, and the future of work, and external shocks to accelerate the changing nature of work. The study also found that these trends were interrelated and could individually and collectively contribute to barriers and opportunities for young people with disabilities in the working world. The Delphi survey we describe will enable participants to generate strategies that specifically address the nine future of work trends that were identified in the recent evidence synthesis [1].

Conceptual approach and Delphi survey overview

Our protocol draws on methods from the field of strategic foresight to obtain future-oriented strategies that can support the employment of young people with disabilities within the context of the future of work. One approach commonly used in strategic foresight is a Delphi survey to obtain insights on uncertain and complex phenomena and develop related solutions. The Delphi

For peer review only - http://bmjopen.bmj.com/site/about/guidelines.xhtml

survey is a participatory process which draws on the knowledge of diverse experts [26 27]. The Delphi survey takes a structured and multistage approach to collect anonymous expert opinions and feedback, facilitate unbiased debate and reach consensus on a phenomenon with the ultimate goal of generating future-oriented recommendations that contribute to policy and programmatic development [27]. The Delphi survey was initially developed by the RAND Corporation in the 1950s for technological forecasting in US military research applications [27] but has been increasingly been applied to policy and programmatic development in a range of sectors including technology, finance, education, and health [28-31]. More recently, online Delphi surveys have become more common and enable participants to complete the study at their own time and pace and enable researchers to collect insights from diverse geographical locations. An online Delphi survey format also ensures anonymity of participants to address biases that emerge in traditional forms of in-person group decision-making and can ensure that data collection is accessible to participants with disabilities and the assistive devices they may require (e.g., completion of Delphi survey using screen readers) [28 32-35].

Sample recruitment

A key element in the design and implementation of a Delphi survey is the selection of Canadian and international participants with subject matter expertise on the employment of young people

For peer review only - http://bmjopen.bmj.com/site/about/guidelines.xhtml

with disabilities or the changing world of work and/or the lived experience of a disability [36]. Subject matter experts recruited for this study will include policymakers, disability employment service providers, educators, clinicians, and futurists. All eligible participants should be above 18 years of age, willing to participate in an open-ended survey where they can provide suggestions on design of innovative strategies to support the employment of young people with disabilities within the context of different future of work trends.

Recruitment will be purposive to ensure we capture a wide range of expertise. Participants will be recruited with the support of our advisory panel and research colleagues who will send direct study invitations to eligible subject matter experts and people with the lived experience of a disability. Additionally, recruitment information will be shared via social media and listservs maintained by the national disability organizations to which our advisory panel are representatives. We will take a snowball approach to recruitment. Participants who complete the Delphi survey will be asked to nominate other potential participants who may be contacted. Currently no established guidelines exist on the number of participants required for a Delphi survey. Researchers utilizing a Delphi approach suggest that sample sizes should be large enough to capture breadth and depth in perspectives but also be manageable for the research team to enable retention across multiple survey rounds. Aligning with past Delphi studies of a similar nature to our protocol [37], we aim to recruit up to 200 participants to enable descriptive analyses required to establish consensus and address potential challenges related to following up with participants [38].

For peer review only

Survey design

The Delphi survey will be implemented in at least two rounds in English or French languages. According to past applications of Delphi surveys, two rounds can be optimal because it limits participant fatigue and dropout which may bias results [39]. The survey was developed based on multiple stages of pilot testing with members of our advisory panel. Pilot testing ensured face validity of questions, use of comprehensible language and that the survey could be completed within 20 minutes. Also, pilot testing ensured that the format was inclusive for participants with different disabilities and could be used in conjunction with assistive devices (e.g., screen readers).

Delphi survey items are developed to foster virtual brainstorming and debate and capture participant insights on specific strategies that can support the employment of young people with disabilities and are relevant to different trends in the future of work. Prompts are included throughout the survey to encourage participants to think strategically about the future and consider how changing conditions could impact young people with disabilities and their need for workplace, community-based and policy supports. The first survey round will be conducted between June-September 2021 and focuses specifically on idea generation and obtaining specific strategies that can support entry and advancement of young people with disabilities in the future of work [40]. Participants will be presented with a description of the trend and provided with additional reading where they can learn more on the topic if they require. A series of questions corresponding to the specific trend will be asked. Participants will be asked to think about the importance of the trend to shaping the long-term employment of people with disabilities. Also, participants will be asked about whether strategies can be implemented within the community,

For peer review only - http://bmjopen.bmj.com/site/about/guidelines.xhtml

workplace or at the policy-level to address the impact of the trend on the employment of young people with disabilities. Two open-ended questions will ask participants to describe specific strategies to address the impact of the trend, and to explain the rationale behind their responses. Participants will be randomly allocated four out of the nine future of work trends identified from past research. We also include a final open-ended question for participants to include any additional reflections on the future of work and its implications for young people living with disabilities.

All open-ended questions from the first round of the Delphi survey will be coded using thematic analysis and an iterative approach. Specifically, we take a constructivist perspective to inductively examine themes that emerge from the data to build an understanding of the specific strategies that can promote employment engagement in the future of work for young adults with disabilities[41 42]. To implement our analytical approach, open-ended survey responses will be reviewed by members of the research team, and a codebook will be developed through several conversations. The codebook will be applied to analyze Delphi survey responses. A primary and secondary coder will conduct a line-by-line coding of responses. Throughout the analytical process, codes and themes emerging from the Delphi survey will be discussed in research team analysis meetings and any inconsistencies will be resolved by consensus. Through our analytical process, a standardized list of strategies for young people with disabilities that are relevant to each trend in the future of work will be generated. A summary of findings will be integrated into

For peer review only - http://bmjopen.bmj.com/site/about/guidelines.xhtml

a short report. Findings will be member checked by our advisory panel to ensure trustworthiness. The study team will ensure that findings are presented anonymously so that participant responses are not biased in subsequent phases of the Delphi survey [43].

The second round of the Delphi survey, administered after six months (March-June 2022), aims to reach consensus across study participants. All participants will be asked to read the brief report that will include findings from the previous round that are aggregated across all study participants as well as a summary of the participant's specific responses. Participants will be asked to complete the second round of the survey while considering findings from the first round. Participants will be presented with the same four trends that they were presented with in round one and the corresponding strategies that were generated. Participants will be asked to rate their level of agreement with the specific strategies that have been proposed for each trend using Likert-style questions (e.g., 1=not at all agree; 5=extremely agree). Additionally, open-ended questions will be posed to participants to ask about potential barriers to implementing strategies and to capture changes to their perspectives on the future of work that could have emerged after the first round of the survey.

The results of the second Delphi survey will be summarized using descriptive statistics [27]. There exists no widely used recommendations on cut-off points that can be used by research to establish consensus in a Delphi survey. Aligning with past applications of the Delphi survey approach, consensus will be reached if at least 70% of participants report agreement on the ability of specific strategy to address a future of work trend [37]. We anticipate that there may be future of work strategies which do not achieve the threshold for consensus. These discordant strategies that may still be effective in promoting employment participation. We will document discordant strategies and share them in our final publications to encourage future research on

For peer review only - http://bmjopen.bmj.com/site/about/guidelines.xhtml

their effectiveness. Open-ended questions will be thematically coded using the same iterative approach that is taken in the analysis of findings in Delphi survey round 1. Following the completion of the second round of the Delphi survey, we anticipate having generated consensus-based policy and programmatic recommendations that can support young people with disabilities that are relevant to the future of work. We will also be able to obtain specific insights into the obstacles that may be faced in implementing and accessing the proposed strategies.

Patient and Public Involvement

Our study takes an integrated knowledge transfer and exchange approach (iKTE) which is defined as a process of two-way exchange between researchers and diverse stakeholders at all phases of the research process including study design and implementation, analysis and synthesis and dissemination to increase research relevancy and evidence uptake and application [44-46]. To implement an iKTE approach in our protocol, we established an advisory panel consisting of young people with lived experience of disability [46] and representatives from national non-profit disability organizations. Advisory panel members have been engaged to inform study design and recruitment and accessibility of data collection approaches. The advisory panel will be asked to help interpret findings that emerge from the first and second rounds of the Delphi. Advisory panel members will also be engaged in the co-creation of study recommendation and knowledge mobilization outputs.

Ethics and dissemination

The research has been cleared by the University of Toronto's Research Ethics Board (#40727).

All data collection and analysis will be performed in accordance with research ethics guidelines and regulations for research on human subjects. Of note, all participants will be presented with

For peer review only - http://bmjopen.bmj.com/site/about/guidelines.xhtml

detailed study material and informed consent will be obtained prior to their participation in the study. Honorariums will be provided to participants with lived experience. All data we collect will be anonymized and stored on a secure server. Our iKTE approach will produce findings that are more relevant to the unique lived experience of young people with disabilities and useable to stakeholders to inform strategies for the future of work [45]. In collaboration with our advisory panel, we will design outputs that are tailored and shared to diverse knowledge users including policy makers, disability employment service providers, employers and people living with disabilities.

DISCUSSION

Young people with disabilities represent a vulnerable labor market subgroup who may require strategies to address the challenges associated with entering and advancing within the world of work. In the future, work is expected to shift the nature and availability of work and, as a result, create new barriers and facilitators to employment participation. The Delphi survey methodology that we present has the potential to contribute to future-oriented insights regarding the specific strategies that can be used by employers, policymakers, and employment service providers to promote the employment of people with disabilities that are resilient to the changing nature of work. Importantly, the strategies produced through the Delphi survey have the potential to

For peer review only - http://bmjopen.bmj.com/site/about/guidelines.xhtml

strengthen present and future socioeconomic pathways to health and quality of life of young people with disabilities [47].

Past studies of young people with disabilities highlight the importance of diverse support strategies to foster employment and career advancement including workplace practices (e.g., job accommodation), labor market policies (e.g., anti-discrimination laws) and community-based programs (e.g., work-integrated learning) [3 12]. Yet, social, political, technological and environmental changes in the future of work will drive changes to workplace and labor market conditions [1 17]. As a result, existing support strategies for people with disabilities will require innovation. In this paper, we describe a Delphi survey protocol which is a well-suited methodology to undertake exploratory research to develop insights on the disability support strategies in the future of work. Findings from our study can be used to update existing policies and program for people with disabilities or inform the design of innovative initiatives that are specifically relevant to the future of work. Past scholars note that Delphi surveys have the advantage of being a rapid approach to collecting and combining expertise from diverse subject matter experts across geographic regions [37]. A Delphi approach may not replace methods which establish causation. Intervention research will be required to test the support strategies uncovered

For peer review only - http://bmjopen.bmj.com/site/about/guidelines.xhtml

in the Delphi study in terms of their ability to foster the long-term employment of young people with disabilities.

A key objective of this Delphi survey will be to facilitate the convergence of future-focused perspectives among participants and forecast support strategies [48]. By administering the survey over two or more rounds, participants will be exposed to the responses of others. In doing so, they will explore their underlying assumptions regarding the future of work which may have informed their responses and compare their perspectives to those held by other subject matter experts [27]. While the focus of our Delphi approach is to establish consensus on future of work support strategies, we will also review strategies that have been proposed where there is a lack of consensus [39]. Additional follow-up research may be required to examine the potential implications of strategies that are recommended by participants and where consensus is not reached to determine utility towards supporting young people with disabilities

Research and practice on the employment of people with disabilities highlight the importance of that multiple perspectives are necessary to identify different barriers and facilitators to participation in paid work and develop corresponding solutions [49]. We will recruit policymakers from different levels of government and labor market specialists and employment service providers from across Canada to capture a range of insights regarding the strategies that

For peer review only - http://bmjopen.bmj.com/site/about/guidelines.xhtml

correspond to different changes in the future of work. Drawing on a diversity of insights can contribute to the development of future-focused wraparound supports which are seen as an important strategy to foster the employment of people with disabilities. Moreover, disability scholars highlight an imperative of ensuring the lived experience to predominate in research on people with disabilities [49 50]. Our study takes an iKTE approach; we created an advisory panel where we integrate lived experience into study design and implementation, data analysis and knowledge mobilization. Through our iKTE approach we will produce findings that are applicable to diverse knowledge users and can be implemented to address different challenges faced by young people with disabilities in the future of work [45 46]. We anticipate that our findings will also have indirect outcomes for advisory panel members and study participants. By participating in the Delphi survey, participants will be encouraged to take a future-focused perspective towards understanding the employment challenges and opportunities faced by young people with disabilities and take a more proactive approach to designing support strategies [51]. It is important to note that while our study is conducted within the context of Canada's labor market and social policy context, it will produce insights that are relevant for the inclusion of

For peer review only - http://bmjopen.bmj.com/site/about/guidelines.xhtml

people living with disabilities in other industrialized countries, especially those that are signatories of the CRPD.

There are some limitations of our Delphi survey. First, for subject matter experts, thinking about work-related challenges and opportunities for people with disabilities and generating support strategies from a future-facing perspective could be difficult. To address this challenge, we have included prompts throughout the survey that encourage participants to consider the relevancy of their responses for the future. Secondly, consistent with past applications of a Delphi survey approach, we will take a purposive sampling strategy to identify diverse subject matter experts and people with the lived experience of a disability. While representativeness of the sample is not a primary goal, we will take steps to ensure depth and breadth in survey responses and the development of recommendations to support young people with disabilities in the future of work [43]. Third, loss to follow-up represents a barrier to establishing consensus and could be a source of bias. Although time and resource intensive, members of the research team will take an active approach to engaging and following up with all participants in the second phase of the Delphi survey to address the potential for dropout.

For peer review only - http://bmjopen.bmj.com/site/about/guidelines.xhtml

RUNNING HEAD: INCLUSION IN THE FUTURE OF WORK

For young people with disabilities, employment represents a critical social determinant of health and quality of life. Yet, many young people report challenges as they enter and advance within the working world. In this protocol paper we describe a Delphi survey approach to generate recommendations and build consensus on future-focused employment support strategies drawn form a diverse group of subject matter experts and people with lived experience of a disability. The evidence to be generated from our study has the potential to be used to inform the design of workplace, community-based and policy-level strategies to ensure that the work in the future will include young people with disabilities.

AUTHOR CONTRIBUTIONS

Arif Jetha: Conceptualization, Methodology, Writing - Original Draft, Funding acquisition

Kay Nasir: Conceptualization, Methodology, Writing - Original Draft

Dwayne Van Eerd: Conceptualization, Methodology, Writing – Review and Editing

Monique Gignac: Conceptualization, Methodology, Writing – Review and Editing

Kathleen Martin Ginis: Conceptualization, Methodology

Emile Tompa: Conceptualization, Methodology, Writing – Review and Editing

For peer review only - http://bmjopen.bmj.com/site/about/guidelines.xhtml

RUNNING HEAD: INCLUSION IN THE FUTURE OF WORK

FUNDING

This work was supported by a research operating grant from Accessibility Standards Canada, Employment and Social Development Canada, Government of Canada (#017323700). The study protocol has undergone peer-review by the funding body. Funding bodies had no role in study design and will not be involved in data collection, data interpretation or manuscript writing.

COMPETING INTERESTS

No authors have competing interests to report related to this research.

AVAILABILITY OF DATA AND MATERIALS

The datasets generated and analyzed in the current study will not be publicly available to protect the privacy of research participants as outlined in the study's research ethics protocol. Those with data requests may contact the corresponding author to learn more about why data cannot be released.

REFERENCES

For peer review only

RUNNING HEAD: INCLUSION IN THE FUTURE OF WORK

1. Jetha A, Shamaee A, Bonaccio S, et al. Fragmentation in the future of work: A horizon scan examining the impact of the changing nature of work on workers experiencing vulnerability. *American Journal of Industrial Medicine* 2021;In press

2. Morris SP, Fawcett G, Brisebois L, et al. A demographic, employment and income profile of Canadians with disabilities aged 15 years and over, 2017. Ottawa, ON: Statistics Canada, 2018.

3. Jetha A, Bowring J, Furrie A, et al. Supporting the transition into employment: a study of Canadian young adults living with disabilities. *Journal of occupational rehabilitation* 2019;29(1):140-49.

4. Statistics Canada. A profile of persons with disabilities among Canadians aged 15 years or older, 2012. Ottawa, ON, 2017. Contract No. 89-654-X.

5. Jetha A, Tucker L, Backman C, et al. Rheumatic disease disclosure at the early career phase and its impact on the relationship between workplace supports and presenteeism. *Arthritis Care & Research* 2021 doi: https://doi.org/10.1002/acr.24620

6. Winn S, Hay I. Transition from school for youths with a disability: Issues and challenges. *Disability & Society* 2009;24(1):103-15.

For peer review only - http://bmjopen.bmj.com/site/about/guidelines.xhtml

7. Sanderson E. Youth transitions to employment: longitudinal evidence from marginalised young people in England. *Journal of Youth Studies* 2020;23(10):1310-29.

8. Arvidsson J, Widén S, Tideman M. Post-school options for young adults with intellectual disabilities in Sweden. *Research and practice in intellectual and developmental disabilities* 2015;2(2):180-93.

9. Scarpetta S, Sonnet A, Manfredi T. Rising youth unemployment during the crisis: How to prevent negative long-term consequences on a generation? . *OECD Social, Employment and Migration Working Papers,* 2010; (No.6. 2010).

10. Nevala N, Pehkonen I, Koskela I, et al. Workplace accommodation among persons with disabilities: A systematic review of its effectiveness and barriers or facilitators. *Journal of occupational rehabilitation* 2015;25(2):432-48.

11. Padkapayeva K, Posen A, Yazdani A, et al. Workplace accommodations for persons with physical disabilities: evidence synthesis of the peer-reviewed literature. *Disability and rehabilitation* 2017;39(21):2134-47.

For peer review only

For peer review only - http://bmjopen.bmj.com/site/about/guidelines.xhtml

12. Jetha A, Shaw R, Sinden AR, et al. Work-focused interventions that promote the labour market transition of young adults with chronic disabling health conditions: a systematic review. *Occupational and environmental medicine* 2019;76(3):189-98.

13. Sorensen G, Dennerlein JT, Peters SE, et al. The future of research on work, safety, health and wellbeing: A guiding conceptual framework. *Social Science & Medicine* 2021;269:113593.

14. Baldwin R. The globotics upheaval: Globalization, robotics, and the future of work. New York: Oxford University Press 2019.

15. Manyika J, Chui M, Miremadi M, et al. A future that works: AI, automation, employment, and productivity. (p. 28), 2017.

16. Acemoglu D, Restrepo P. Robots and jobs: Evidence from US labor markets. *Journal of Political Economy* 2020;128(6):2188-244.

17. World Economic Forum. The future of jobs report 2020. Geneva: World Economic Forum., 2021.

18. Lund S, Madgavkar A, Manyika J, et al. The postpandemic econonmy: The future of work after COVID-19: McKinsey Global Institute, 2021.

For peer review only - http://bmjopen.bmj.com/site/about/guidelines.xhtml

For peer review only

19. Autor D, Mindell D, Reynolds E. The work of the future: Building better jobs in an age of intelligent machines, 2020.

20. Acemoglu D, Restrepo P. Low-skill and high-skill automation. *Journal of Human Capital* 2018;12(2):204-32.

21. Benach J, Benavides FG, Platt S, et al. The health-damaging potential of new types of flexible employment: a challenge for public health researchers. *American journal of public health* 2000;90(8):1316-7. doi: 10.2105/ajph.90.8.1316 [published Online First: 2000/08/11]

22. Mann DR, Wittenburg DC. Starting behind: Wage and employment differentials between young adults with and without disabilities. *Journal of Disability Policy Studies* 2015;26(2):89-99.

23. Bonaccio S, Connelly CE, Gellatly IR, et al. The participation of people with disabilities in the workplace across the employment cycle: Employer concerns and research evidence. *Journal of Business and Psychology* 2020;35(2):135-58.

24. An act to ensure a barrier-free Canada. Bill C-81, 2018.

25. United Nations. Convention on the Rights of Persons with Disabilities (CRPD), 2019.

For peer review only

26. Mozuni M, Jonas W. An introduction to the morphological Delphi method for design: A tool for future-oriented design research. *She Ji: The Journal of Design, Economics, and Innovation* 2017;3(4):303-18.

27. Hasson F, Keeney S, McKenna H. Research guidelines for the Delphi survey technique. *Journal of advanced nursing* 2000;32(4):1008-15.

28. Aichholzer G. The delphi method: Eliciting experts' knowledge in technology foresight. Interviewing experts: Springer 2009:252-74.

29. Kozak M, Iefremova O. Implementation of the Delphi technique in finance. *e-Finanse: Financial Internet Quarterly* 2014;10(4):36-45.

30. Nworie J. Using the Delphi technique in educational technology research. *TechTrends* 2011;55(5):24-30.

31. Beech B. The Delphi approach: recent applications in health care. *Nurse Researcher (through 2013)* 2001;8(4):38.

32. Hong SG, Trimi S, Kim DW, et al. A Delphi study of factors hindering web accessibility for persons with disabilities. *Journal of Computer Information Systems* 2015;55(4):28-34.

For peer review only - http://bmjopen.bmj.com/site/about/guidelines.xhtml

33. Helmer-Hirschberg O. Analysis of the future: The Delphi method. Santa Monica: Rand Corporation 1967.

34. Revez A, Dunphy N, Harris C, et al. Beyond Forecasting: Using a Modified Delphi Method to Build Upon Participatory Action Research in Developing Principles for a Just and Inclusive Energy Transition. *International Journal of Qualitative Methods* 2020;19 doi: https://doi.org/10.1177/1609406920903218

35. Dayé C. How to train your oracle: The Delphi method and its turbulent youth in operations research and the policy sciences. *Social Studies of Science* 2018;48(6):846-68.

36. Belton I, MacDonald A, Wright G, et al. Improving the practical application of the Delphi method in group-based judgment: a six-step prescription for a well-founded and defensible process. *Technological Forecasting and Social Change* 2019;147:72-82.

37. Hsu C-C, Sandford BA. The Delphi technique: making sense of consensus. *Practical Assessment, Research, and Evaluation* 2007;12(1):10.

38. Keller J, Heiko A. The influence of information and communication technology (ICT) on future foresight processes—Results from a Delphi survey. *Technological Forecasting and Social Change* 2014;85:81-92.

For peer review only - http://bmjopen.bmj.com/site/about/guidelines.xhtml

39. Gossler T, Sigala IF, Wakolbinger T, et al. Applying the Delphi method to determine best practices for outsourcing logistics in disaster relief. *Journal of Humanitarian Logistics and Supply Chain Management* 2019;9(3):438-74.

40. Arif Jetha AS. Fragmentation in the future of work. Nine trends that may shape the future of work and what they mean for vulnerable workers, 2021.

41. Miles M, Huberman A, Saldaña J. Qualitative data analysis: A methods sourcebook.: Sage publications 2018.

42. Denzin N, Lincoln Y. The Sage handbook of qualitative research:: Sage 2011.

43. Asselin M, Harper M. Revisiting the Delphi technique: implications for nursing professional development. *Journal for Nurses in Professional Development* 2014;30(1):11-15.

44. Dew A, Boydell KM. Knowledge translation: Bridging the disability research-to-practice gap. *Research and Practice in Intellectual and Developmental Disabilities* 2017;4(2):142-57.

45. Rogers JD, Martin FH, Force NKTT. Knowledge translation in disability and rehabilitation research: lessons from the application of knowledge value mapping to the case of accessible currency. *Journal of Disability Policy Studies* 2009;20(2):110-26.

For peer review only

For peer review only - http://bmjopen.bmj.com/site/about/guidelines.xhtml

46. Van Eerd D, Saunders R. Integrated knowledge transfer and exchange: An organizational approach for stakeholder engagement and communications. *Scholarly and Research Communication* 2017;8(1)

47. Ahonen EQ, Fujishiro K, Cunningham T, et al. Work as an inclusive part of population health inequities research and prevention. *American journal of public health* 2018;108(3):306-11.

48. US Department of Health and Human Services Public Health Services Agency for Health Care Policy and Research. Acute Pain Management: Operative or Medical Procedures and Trauma. . Rockville: Acute Pain Management Guideline Panel 1992.

49. Bonaccio S, Connelly CE, Fisher SL, et al. Beyond managing research partnerships: Partnered research as an integrated methodological approach. *Industrial and Organizational Psychology* 2018;11(4):613-19.

50. Harpur P. Nothing about us without us: The UN convention on the rights of persons with disabilities. Oxford Research Encyclopedia of Politics2017.

51. van der Heijden K. Scenarios, strategy and the strategy process. *Global Business Network Presearch, Provoking Strategic Conversation* 1997;1(1)

For peer review only - http://bmjopen.bmj.com/site/about/guidelines.xhtml

For peer review only

For peer review only - http://bmjopen.bmj.com/site/about/guidelines.xhtml
